# Artificial intelligence-based iliofemoral deep venous thrombosis detection using a clinical approach

**DOI:** 10.1038/s41598-022-25849-0

**Published:** 2023-01-18

**Authors:** Jae Won Seo, Suyoung Park, Young Jae Kim, Jung Han Hwang, Sung Hyun Yu, Jeong Ho Kim, Kwang Gi Kim

**Affiliations:** 1 Department of Health Sciences and Technology, GAIHST, Gachon University, Incheon, 21999 Republic of Korea; 2 Department of Radiology, Gil Medical Center, Gachon University College of Medicine, Incheon, 21565 Republic of Korea; 3 Department of Biomedical Engineering, Gil Medical Center, Gachon University, Incheon, 21565 Republic of Korea

**Keywords:** Image processing, Machine learning, Diagnosis, Medical imaging

## Abstract

Early diagnosis of deep venous thrombosis is essential for reducing complications, such as recurrent pulmonary embolism and venous thromboembolism. There are numerous studies on enhancing efficiency of computer-aided diagnosis, but clinical diagnostic approaches have never been considered. In this study, we evaluated the performance of an artificial intelligence (AI) algorithm in the detection of iliofemoral deep venous thrombosis on computed tomography angiography of the lower extremities to investigate the effectiveness of using the clinical approach during the feature extraction process of the AI algorithm. To investigate the effectiveness of the proposed method, we created synthesized images to consider practical diagnostic procedures and applied them to the convolutional neural network-based RetinaNet model. We compared and analyzed the performances based on the model’s backbone and data. The performance of the model was as follows: ResNet50: sensitivity = 0.843 (± 0.037), false positives per image = 0.608 (± 0.139); ResNet152 backbone: sensitivity = 0.839 (± 0.031), false positives per image = 0.503 (± 0.079). The results demonstrated the effectiveness of the suggested method in using computed tomography angiography of the lower extremities, and improving the reporting efficiency of the critical iliofemoral deep venous thrombosis cases.

## Introduction

Deep venous thrombosis (DVT) most commonly develops in the lower extremities and can cause complications that raise mortality and decrease the quality of life^1^. The treatment and long-term prognosis of lower-extremity DVT depend on an accurate and timely diagnosis^2^. However, owing to the absence of a radiologist on duty, diagnosis might be delayed. Because the clinical symptoms and signs have low specificity for the diagnosis of DVT, imaging workup is necessary to confirm or exclude the diagnosis^3^.

Owing to intra- and inter-observer variability, medical imaging analysis is typically performed manually; it places a burden on the radiologist and increasing the risk of misdiagnosis. Because of the drawbacks of manual analysis, convolutional neural network (CNN)-based artificial intelligence (AI) algorithms have been used in the medical imaging field as a computer-aided diagnosis (CAD) system tool^4^. Moreover, some studies proposed AI techniques to improve the diagnostic performance by fusing the clinical information or practical diagnostic procedures, and demonstrated the benefit of aggregated clinical approaches^5–7^.

Imaging modalities for DVT diagnosis include ultrasonography (US), computed tomography angiography of the bilateral lower extremities (LECTA), magnetic resonance imaging (MRI), and catheter venography. To overcome the limitations of the DVT manual analysis, studies have been conducted using various imaging modalities and have shown the potential and efficiency of an AI-based CAD system for DVT diagnosis^8–12^. Among the image modalities, LECTA was found to be more advantageous—it provided more objective images than US; it is easily accessible and frequently used to provide information about extravascular tissues in the bilateral lower extremities and abdominopelvic region. In the clinical setting, to accurately decide on DVT in LECTA, adjacent slices should be considered rather than one CT slice with the suspicious existence of the lesion. As preliminary studies, we conducted two kinds of DVT diagnosis based on CNN models in LECTA. The first study aimed to investigate quantitative differences between with region of deep venous with and without DVT by classifying the region of deep vein. This formative study indicated that the CNN model can extract useful features that can distinguish the region containing DVT from other regions^12^. However, the result of the study did not include information about localization of the DVT in LECTA. The next study explored the possibility of applying CNN-based detection models for DVT detection which contains localization of the DVT in LECTA^10^. This previous study did not consider the clinical diagnostic approach. Therefore, the current study aimed to the use of CNN models for improving diagnostic performance by dealing with the clinical process of detecting iliofemoral DVT on LECTA in AI algorithm. In this study, we demonstrated that the AI model extracted significant features for detecting DVT by applying clinical approach, as though clinical diagnosis to DVT considers the upper and lower image slices.

## Methods

### Data collection

**Figure 1:**
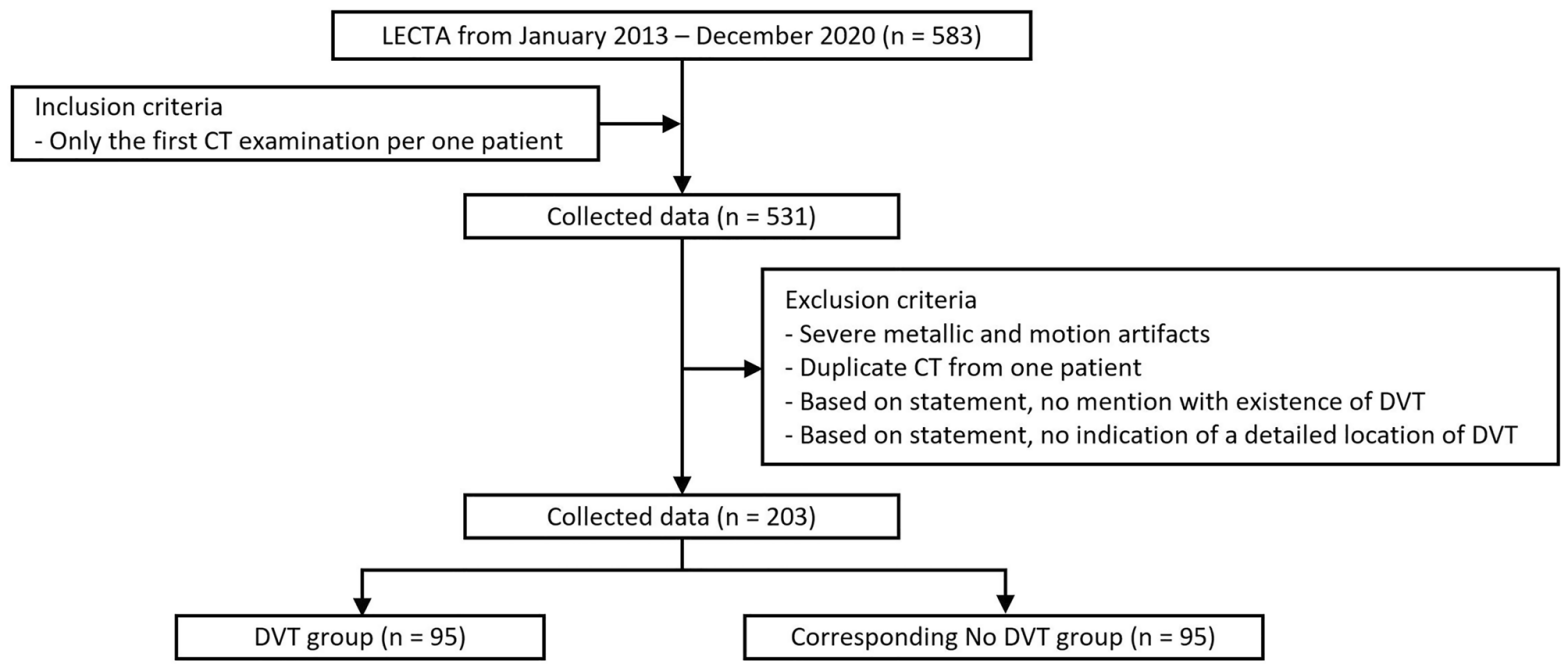
Flowchart of data selection.

The institutional review board of Gachon University Gil Medical Center (IRB Number: GAIRB2021-225) approved this study; the requirement of informed consent was waived for this study populations because of the study design’s retrospective nature. All experimental protocols were performed in accordance with the relevant guidelines and regulations in compliance with the Declaration of Helsinki. The picture archiving and communication system database was searched for LECTA examinations conducted at Gil Medical Center between January 2013 and December 2020, and 583 consecutive LECTA examinations were identified. When a patient underwent multiple LECTA examination sessions, only the first LECTA scan session of the patient was considered for this study. Patients with motion or metallic artifacts were excluded. Additionally, cases without a detailed mention of the presence or absence of iliofemoral DVT in the radiologic report were excluded. Consequently, 380 LECTA examination sets were disqualified. Among them, 95 sets with iliofemoral DVT on the radiological report were grouped as the “DVT” group. Likewise, 95 LECTAs without iliofemoral DVT on the radiological report were systematically gathered and grouped as “no DVT” group (Fig. 1).

### LECTA image acquisition

LECTA was performed using 128-slice scanners (Somatom Definition Flash and Somatom Definition Edge; Siemens Healthcare, Erlangen, Germany). The acquisition range was determined from the T12 vertebra to the lower end of the feet. Images were obtained 4 min after a 2 mL/kg of non-ionic contrast agent intravenous injection (Bonorex 350; Central Medical Service, Seoul, Republic of Korea). Axial images were obtained in digital imaging and communication in medicine (DICOM) format with a 5-mm slice thickness and 5-mm slice interval.

### Data preprocessing

To enhance the contrast of the blood vessels from the background, the window width was set to 400 Hounsfield units (HU) and the window level to 140 HU. The region of interest (ROI) for the blood vessels was collected using the image set with the corresponding values in the DICOM image. The average size of the ROIs for the collected veins was 7.755 (± 2.594) × 7.801 (± 2.511) pixels, indicating that the veins occupied an insignificant proportion of the overall axial image size (512 × 512 pixels). Consequently, we set the data pixel spacing to the minimum value (0.695 mm). The pixel spacing range was 0.695–0.977 mm. For the same reason, randomly extracted patch images of 128 × 128 pixels of the areas surrounding the iliofemoral vein from the DICOM images limited the area of detection. A total of 6965 patch images (3706 and 3259 images from the DVT and no DVT groups, respectively) are generated from 190 of patients. We ran a five-fold cross-validation to assess the model’s performance. The folds were divided by the number of patients. For training the models, we used average 4179(± 183.458) patches from 114 patients as a train set and average 1471.2(± 184.671) patches from 38 patients as a validation set. A test set for performance assessment is composed of average 1314.8(± 109.523) patches generated from 38 patients.

**Figure 2:**
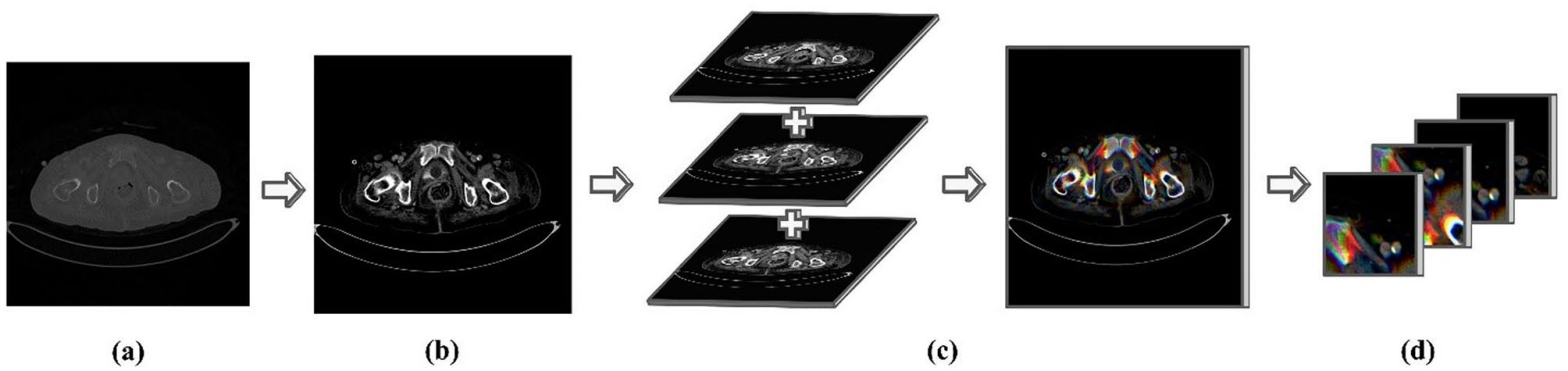
The process of generating synthesized three-slice data. (**a**) An original raw image; (**b**) An image-uniformed pixel spacing and set to a window width of 140 Hounsfield units (HU) and window level of 400 HU; (**c**) A synthesized image; and (**d**) A patch unit image.

To compare the performance of one slice image with that of the three slices data (one image, one upper image, and one lower image), we synthesized an image using three continuous LECTA images while considering the characteristics of clinical diagnosis (Fig. 2).

### Deep learning based on convolutional neural networks

We chose the CNN-based RetinaNet model to detect iliofemoral DVT because it has the advantages of time efficiency and high accuracy based on its loss function and structure. RetinaNet is a deep learning-based one-stage detection model that uses a focal loss function, and has demonstrated strong performance in addressing the foreground–background class imbalance, which is the main drawback of one-stage object detectors^13^. The RetinaNet has a feature pyramid network combined with the ResNet backbone^14^. The feature pyramid network has been applied and used in many detection models in medical imaging because it exhibits a high level of detection performance with minimal resource requirements for computation; it uses CNN to extract four different multiscale feature maps from one image^15–17^. The RetinaNet uses a pyramidal structure to construct the multiscale feature maps that the ResNet backbone network extracts. The RetinaNet structure has two distinct subnetworks using a region-proposal-based network from the feature map extracted by each pyramid layer. One performs regression for localization to the bounding box of the target object task, while the other performs object classification. Regarding time consumption, RetinaNet is a one-stage detector that accomplishes two tasks concurrently for high performance and efficiency.

The study was performed in Python 3.6.12 (Python Software Foundation, Wilmington, DE) using Keras 2.2.5 frameworks (Keras Global Limited, London, United Kingdom) on an Ubuntu 14.04 operating system (London, United Kingdom) with two NVIDIA Tesla P100 graphics processing units (GPUs; NVIDIA Corporate, Santa Clara, CA) and 512 GB of random access memory. The hyper-parameters are manually set as follows: 16 batch size, 100 epochs and a 0.0001 learning rate. The hyper-parameters are set to 16 batch size, 100 epochs and 0.0001 learning rate. We set the learning rate to decrease by a factor of 0.1 if the validation loss did not decrease for 15 epochs.

### Performance assessment

The performance assessment was conducted using the test data from 38 cases that were not used as the training sets from each fold. The intersection over union (IOU) refers to an evaluation index based on the overlap between the two areas. In this study, the two areas stand for the ground truth (GT), which is the ROI labeled by radiologists, and the prediction area derived from the models. The IOU value threshold was set at 0.1. The bounding box that the model predicted was treated as a true positive (TP) if the IOU value calculated from the two areas was found to be greater than or equal to 0.1. If the value was found to be less than 0.1, the predicted box was considered a false positive (FP). A false negative (FN) was declared if the model’s GT prediction area was absent.

The sensitivity (Sn and recall), FPs per image (FPPI), and precision were calculated using the model’s evaluation indicators. The average precision (AP) refers to the area under the precision-recall curve. The following equations define them:

$$Sn=\frac{TP}{TP+FN}$$

$$FPPI=\frac{FP}{the \; number \; of \; images}$$

$$Precision=\frac{TP}{TP+FP}$$

$$mAP=\frac{AP}{the \; number \; of \; classes}$$

## Results

**Figure 3:**
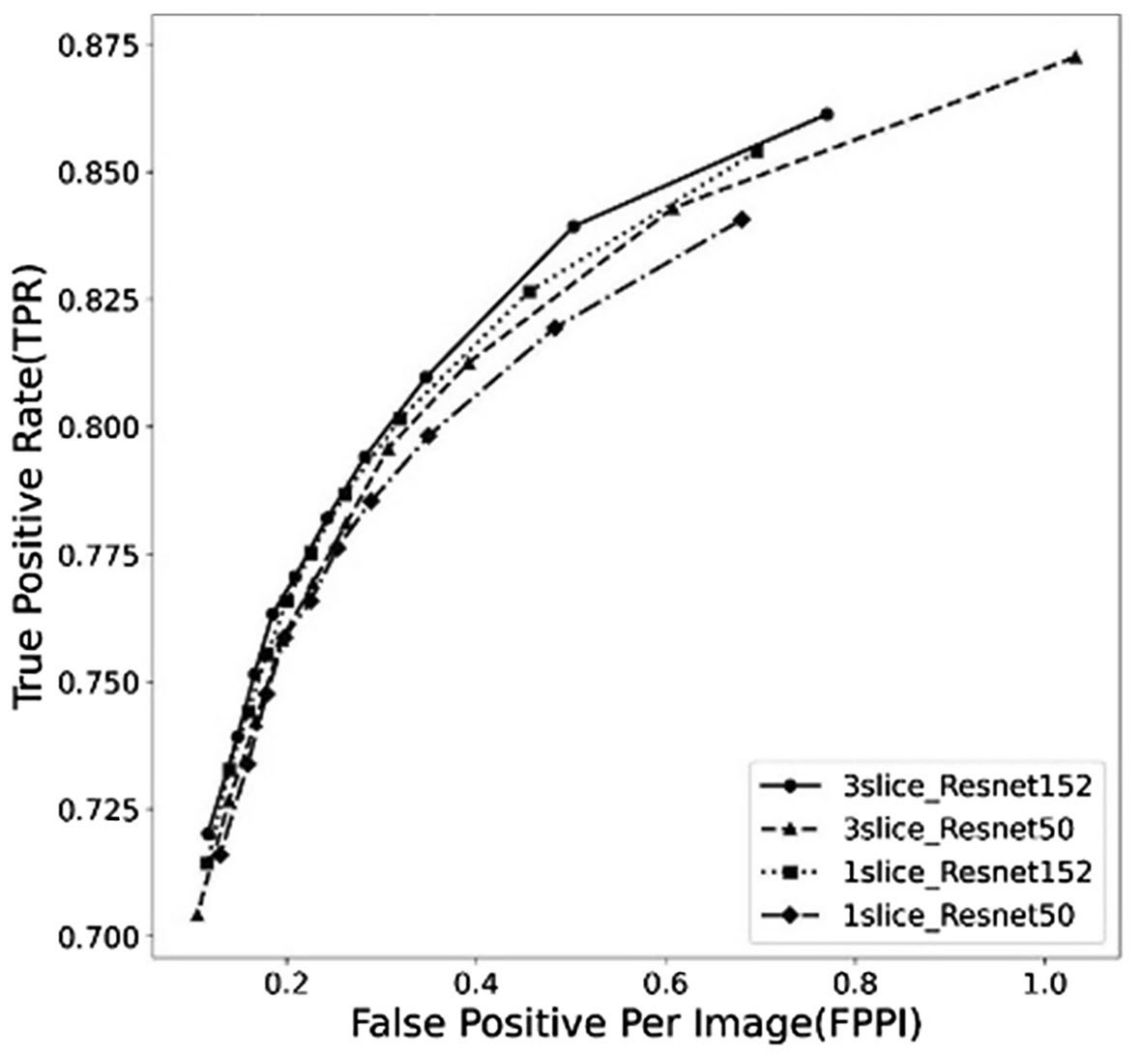
Free response operating characteristic curves of results from the models using proposed three slice data and one slice data based on ResNet152 and ResNet50 backbones, respectively.

**Figure 4:**
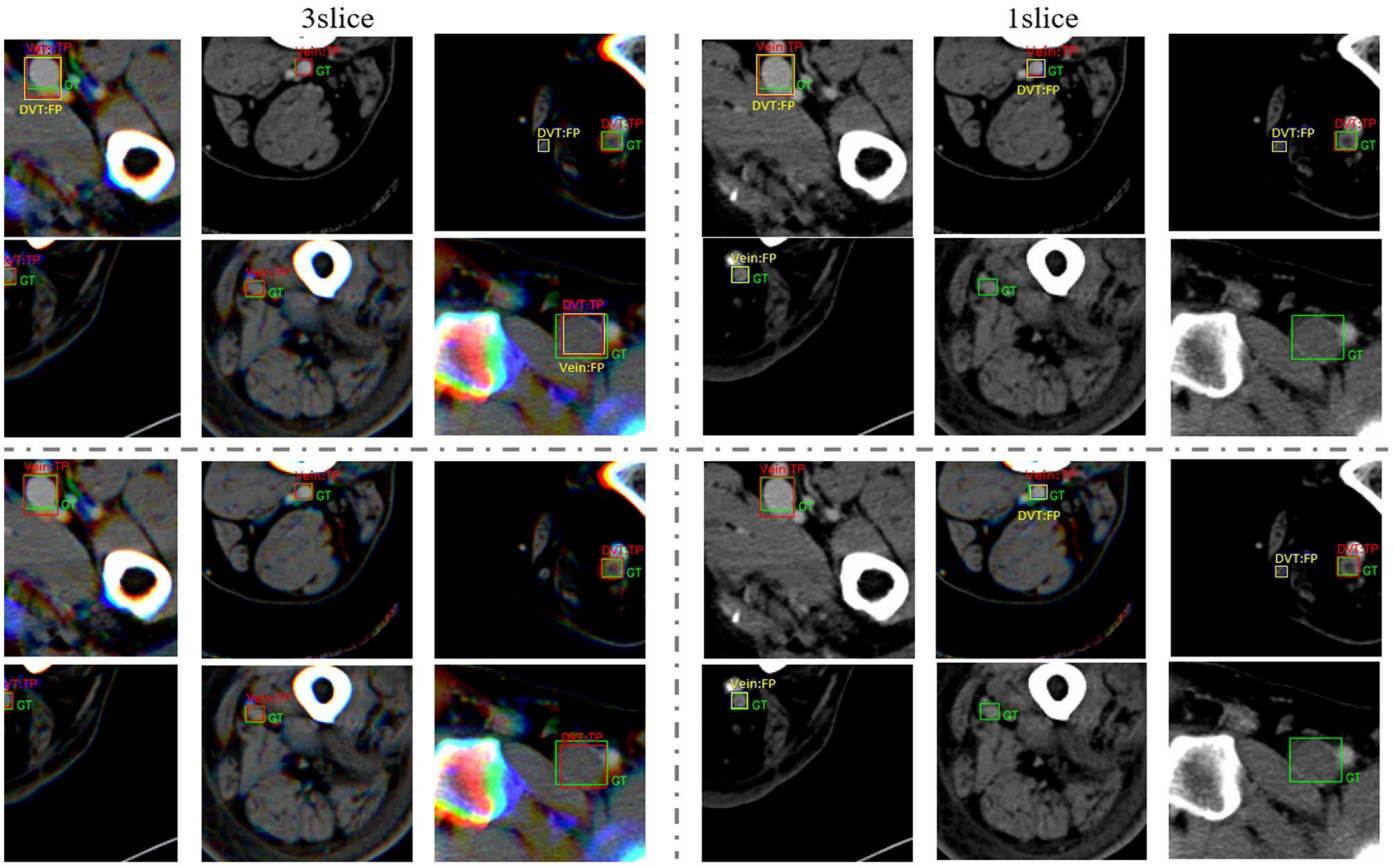
The example of results-image patches from model’s each backbone (ResNet50 and ResNet152) and each used data (3 slices and one slice). The red rectangle box means true positive result from the model. The green rectangle box is the ground truth, and the yellow rectangle box indicates a false positive result from the model.

Table 1 displays the average results of the performance indicators (Sn, FPPI, precision, and mAP) from test data of each fold according to the number of slices (synthesized three slices and only one slice) and backbones (ResNet50 and ResNet152) based on the 0.1 IOU value threshold and 0.1 confidence threshold. The model-based ResNet152’s backbone performances using the three suggested synthesized slices yielded 0.839 (± 0.031) Sn, 0.503 (± 0.079) FPPI, 0.650 (± 0.038) precision, and 0.806 (± 0.034) mAP. In the model based on the ResNet50 backbone using the same proposed data, the performances approached 0.843 (± 0.037) Sn, 0.608 (± 0.139) FPPI, 0.610 (± 0.061) precision, and 0.807 (± 0.040) mAP. For the Sn and mAP values, the model with three slices based on the ResNet50 backbone approached 0.843 (± 0.037) and 0.807 (± 0.040) as the highest scores (Table 2). However, based on the ResNet152 backbone, the model with one slice performed the best with 0.456 (± 0.093) FPPI and 0.670 (± 0.047) precision values (Table 3, Fig. 4). Figure 3 shows the free response operating characteristic (FROC) curves based on the Sn and FPPI values of each model result.

**Table 1:** The performance results from each model.

Models	Sn (95% CI)	FPPI (95% CI)	Precision (95% CI)	mAP (95% CI)
**3 slices**				
ResNet152	0.839 (0.808–0.870)	0.503 (0.424–0.582)	0.650 (0.612–0.688)	0.806(0.772–0.841)
ResNet50	0.843* (0.806–0.880)	0.608 (0.469–0.747)	0.610 (0.549–0.671)	0.807* (0.767–0.847)
**1 slice**				
ResNet152	0.826 (0.799–0.853)	0.456* (0.363–0.549)	0.670* (0.623–0.717)	0.802 (0.773–0.830)
ResNet50	0.819 (0.788–0.850)	0.483 (0.414–0.552)	0.654 (0.621–0.687)	0.784 (0.749–0.819)

*CI* confidence interval, *Sn* sensitivity, *FPPI* false positive per image, *mAP* mean average precision.

*Highest values of comparison parameters.

**Table 2:** Comparison of performance according to data.

Performance	3—slices	1—slice
Sn (95% CI)	0.841* (0.839–0.843)	0.823 (0.819–0.826)
FPPI (95% CI)	0.556 (0.503–0.608)	0.470* (0.456–0.483)
Precision (95% CI)	0.630 (0.610–0.650)	0.662* (0.654–0.670)
mAP (95% CI)	0.807* (0.806–0.807)	0.793 (0.784–0.802)

*CI* confidence interval, *Sn* sensitivity, *FPPI* false positive per image, *mAP* mean average precision.

*Highest values of comparison parameters.

**Table 3:** Comparison of performance according to models.

Performance	ResNet152	ResNet50
Sn (95% CI)	0.833* (0.826–0.839)	0.831 (0.819–0.843)
FPPI (95% CI)	0.480* (0.456–0.503)	0.546 (0.483–0.608)
Precision (95% CI)	0.660* (0.650–0.670)	0.632 (0.610–0.654)
mAP (95% CI)	0.804* (0.802–0.806)	0.796 (0.784–0.807)

*CI* confidence interval, *Sn* sensitivity, *FPPI* false positive per image, *mAP* mean average precision.

*Highest values of comparison parameters.

## Discussion

To detect iliofemoral DVT in LECTA, this study used deep learning-based AI techniques. To further reflect information about the periphery based on the z-axis of the lesion, we produced data by synthesizing three successive images centered on the lesion. To verify the validity of the three generated slices of the synthesized data, we compared their performance with the data that only included the slice identified as having a lesion. According to Table 2, the two models that used the suggested synthesized data performed better in terms of Sn and mAP values. However, the models based on one-slice data outperformed those based on three-slices data. By adding axis-z-based peripheral information to one image, despite an increase in FP cases, the detection rate for veins was higher. Additionally, because the location of the veins in the muscle and bone are relatively similar, we inferred that the ResNet152-based model fitted with more parameters performed better in all indicators based on the depth of the proposed models.

Figure 4 shows the images of the detection result for each backbone based on the type of data used. As shown by the Sn and FPPI values for each model shown in Table 1, the results of using three slice images in the patch image of the same region showed that the number of TP and FP were higher than that of the model using one slice image.

**Figure 5:**
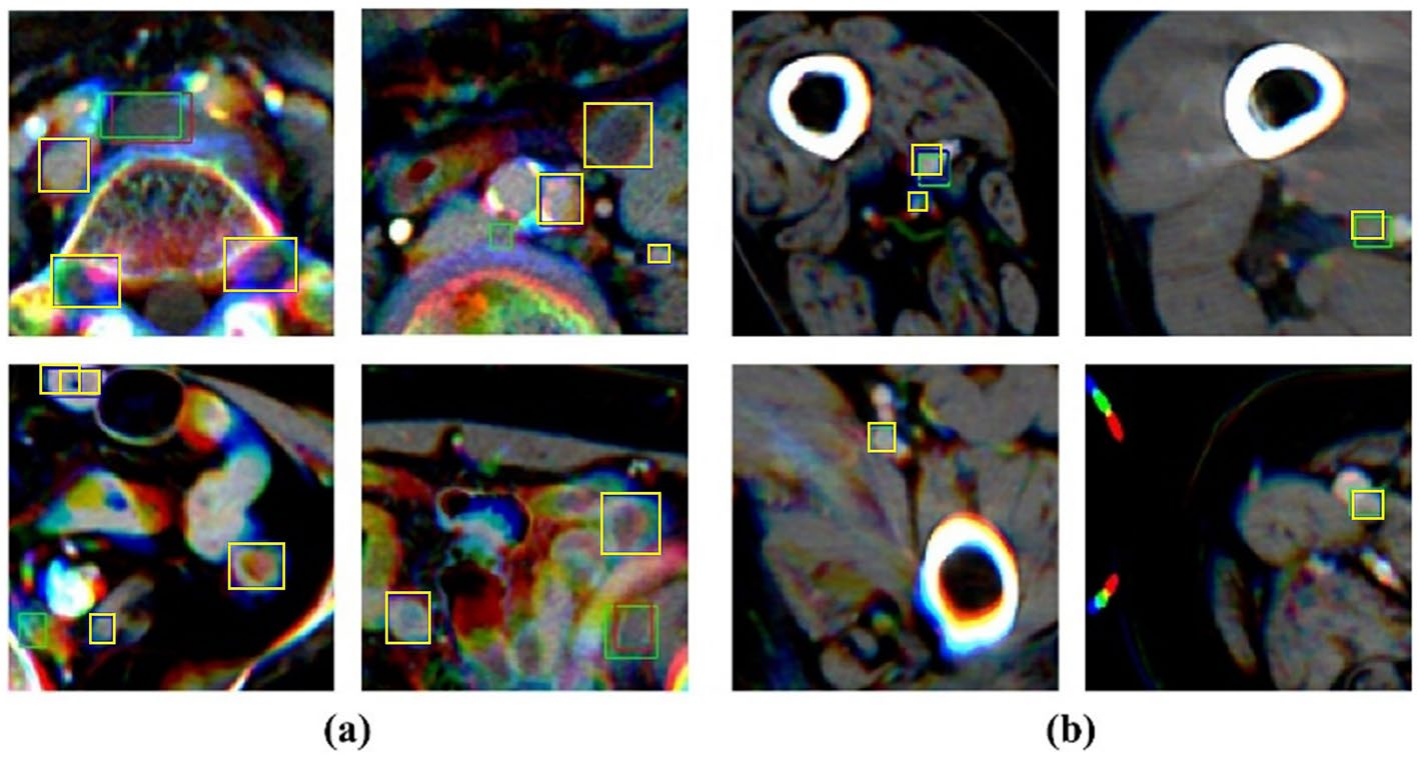
The example of false positive cases image patches from the ResNet50 backbone-based mode with three slices data. The green rectangle box indicates the ground truth, and the yellow rectangle box indicates FP results. **(a)** represents the cases of the wrong detection and **(b)** represents the cases of success for localization but failure to classification.

Figure 5 shows examples of the FP case. As shown in Fig. 5a, most FP cases occurred in areas with similar DVT shapes and pixel intensities. Because of the relatively small area of the generated patch unit that was compared with the entire image, it lacked the peripheral information to confirm the site of analysis as venous. Therefore, we assume that this small area patch led the model to mispredict the DVT for objects with a similar shape. Figure 5b shows a case of successful localization and unsuccessful classification. By creating patch unit images of the region around the iliofemoral veins, we collected data from various backgrounds. The generated image has a background similar to the shape and position of muscles and bones, particularly in the thigh range, which comprises most input data. This aids the model’s ability to locate the objects. However, the number of cases used in this experiment was insufficient to extract the features of various types of DVT and healthy veins.

## Conclusions

The CNN-based models outperformed the one-slice images in detecting iliofemoral DVT on LECTA using the proposed synthesized images. From the results, we demonstrated AI, reflecting the practical process, enables a more accurate diagnosis of DVT detection for LECTA. Our work allows the radiologists to achieve a more accurate diagnosis by utilizing this proposed AI algorithm which presents a location with a probability of the existence of DVT. The radiologists can primarily confirm the locations suggested by the AI model in CT volume data, comprised of numerous slices images. It can lead to improving the reading efficiency of radiologists and reducing the burden on them.

A few research is considered future works. First, it is essential to extend the detection ranges to the infrapopliteal vein for investigating DVT. This research is limited to the iliofemoral vein; hence, the infrapopliteal deep vein was excluded from this study because of its minimal diameter and inconstant location. The infrapopliteal DVT, particularly in high-risk patients, has clinical and diagnostic value because it can spread to the iliofemoral vein^18^. Therefore, it could be possible to increase the DVT detection rate of physicians by developing an AI model with more broaden detection range than this limited range. Second, the result from the proposed AI model should be compared with the diagnosis given by a radiologist to demonstrate the practical advantages of the model. This study attempted to prove the positive aspects of the proposed method by comparing and analyzing the results by applying the method to different AI algorithms for the effect of the proposed algorithm on the AI model. Hence, the research explores the benefit of this AI model for clinical diagnosis as a CAD system.

## Data Availability

The LECTA data used to support the findings of this study are available upon request from the corresponding authors.

## References

[1] Enden T (2012). Long-term outcome after additional catheter-directed thrombolysis versus standard treatment for acute iliofemoral deep vein thrombosis (the CaVenT study): A randomised controlled trial. Lancet.

[2] Schaefer JK, Jacobs B, Wakefield TW, Sood SL (2017). New biomarkers and imaging approaches for the diagnosis of deep venous thrombosis. Curr. Opin. Hematol..

[3] Pollack CV (2011). Clinical characteristics, management, and outcomes of patients diagnosed with acute pulmonary embolism in the emergency department: Initial report of EMPEROR (Multicenter Emergency Medicine Pulmonary Embolism in the Real World Registry). J. Am. Coll. Cardiol..

[4] Jiang J, Trundle P, Ren J (2010). Medical image analysis with artificial neural networks. Comput. Med. Imaging Graph.

[5] Huang SC, Pareek A, Seyyedi S, Banerjee I, Lungren MP (2020). Fusion of medical imaging and electronic health records using deep learning: A systematic review and implementation guidelines. Npj Digit. Med..

[6] AlGhamdi M, Abdel-Mottaleb M (2021). DV-DCNN: Dual-view deep convolutional neural network for matching detected masses in mammograms. Comput. Methods Progr. Biomed..

[7] Gao XHW, Hui R, Tian ZM (2017). Classification of CT brain images based on deep learning networks. Comput. Methods Prog. Biol..

[8] Huang C (2019). Fully automated segmentation of lower extremity deep vein thrombosis using convolutional neural network. Biomed. Res. Int..

[9] Kainz B (2021). Non-invasive diagnosis of deep vein thrombosis from ultrasound imaging with machine learning. Npj Digit. Med..

[10] Seo JW, Kim YJ, Kim KG (2021). Deep vein thrombosis detection based on deep learning for CT images. Int. Conf. Inf. Commun..

[11] Sun C (2021). Deep learning for accurate segmentation of venous thrombus from black-blood magnetic resonance images: A multicenter study. Biomed. Res. Int..

[12] Hwang JH (2022). Comparison between deep learning and conventional machine learning in classifying iliofemoral deep venous thrombosis upon CT venography. Diagnostics.

[13] Lin TY, Goyal P, Girshick R, He KM, Dollar P (2020). Focal loss for dense object detection. IEEE Trans. Pattern Anal..

[14] Lin, T.-Y. *et al.* In *Proceedings of the IEEE conference on computer vision and pattern recognition* 2117–2125.

[15] Guan B, Yao J, Zhang G, Wang X (2019). Thigh fracture detection using deep learning method based on new dilated convolutional feature pyramid network. Pattern Recogn. Lett..

[16] Yang R, Yu Y (2021). Artificial convolutional neural network in object detection and semantic segmentation for medical imaging analysis. Front. Oncol..

[17] Yang M (2020). Deep retinanet for dynamic left ventricle detection in multiview echocardiography classification. Sci. Program..

[18] Robert-Ebadi H, Righini M (2017). Management of distal deep vein thrombosis. Thromb. Res..

